# Forest Canopy Gap Distributions in the Southern Peruvian Amazon

**DOI:** 10.1371/journal.pone.0060875

**Published:** 2013-04-15

**Authors:** Gregory P. Asner, James R. Kellner, Ty Kennedy-Bowdoin, David E. Knapp, Christopher Anderson, Roberta E. Martin

**Affiliations:** 1 Department of Global Ecology, Carnegie Institution for Science, Stanford, California, United States of America; 2 Department of Geographical Sciences, University of Maryland, College Park, Maryland, United States of America; Lakehead University, Canada

## Abstract

Canopy gaps express the time-integrated effects of tree failure and mortality as well as regrowth and succession in tropical forests. Quantifying the size and spatial distribution of canopy gaps is requisite to modeling forest functional processes ranging from carbon fluxes to species interactions and biological diversity. Using high-resolution airborne Light Detection and Ranging (LiDAR), we mapped and analyzed 5,877,937 static canopy gaps throughout 125,581 ha of lowland Amazonian forest in Peru. Our LiDAR sampling covered a wide range of forest physiognomies across contrasting geologic and topographic conditions, and on depositional floodplain and erosional *terra firme* substrates. We used the scaling exponent of the Zeta distribution (**λ**) as a metric to quantify and compare the negative relationship between canopy gap frequency and size across sites. Despite variable canopy height and forest type, values of **λ** were highly conservative (**λ**_ mean_  = 1.83, s  = 0.09), and little variation was observed regionally among geologic substrates and forest types, or at the landscape level comparing depositional-floodplain and erosional *terra firme* landscapes. **λ**-values less than 2.0 indicate that these forests are subjected to large gaps that reset carbon stocks when they occur. Consistency of **λ**-values strongly suggests similarity in the mechanisms of canopy failure across a diverse array of lowland forests in southwestern Amazonia.

## Introduction

Canopy gaps are openings in forest canopies caused by structural failures ranging in size from individual branch loss to multiple treefalls. At a given time, the spatial variability of canopy gaps expresses patterns of mortality and physical damage, in addition to subsequent gap filling that occurs via regrowth and secondary succession. Termed *static* gaps, these openings in the canopy provide insight to the spatial variation in carbon stocks, habitat, and many other forest structural characteristics and functional processes [1]–[7].

Although size-frequency distributions of canopy gaps provide a unique view of the disturbance and recovery regimes of forests, for the most part, their spatial and temporal variation remains very poorly understood, due to the unmet requirement of measuring large numbers of canopy gaps to develop distributional information. A challenge for tropical forest ecology rests in the precise spatial and temporal frequency of gap formation. For decades, forest gap formation, and thus disturbance regimes, have been assessed from opposing vantage points. Field plots have been used to estimate rates and patterns of gap-phase dynamics at a local scale, usually of one hectare or less [7], [8]. In contrast, large disturbances – those driven by humans such as logging as well as natural events like forest canopy blowdowns – have been mapped over large geographies using satellite sensors [9], [10]. Quantifying the continuum of disturbance sizes and frequencies between these extremes remains a major challenge, particularly in the context of mapping the geography of forest disturbance regimes [11].

Recently, an airborne three-dimensional (3-D) laser measurement approach called Light Detection and Ranging (LiDAR) has been used to overcome the challenges of distinguishing millions of canopy gaps at large spatial scales [12], [13], opening the door for an improved understanding of canopy-gap size-frequency distributions. Using airborne LiDAR, Kellner and Asner [12] discovered a surprising level of similarity among canopy gap-size frequency distributions in Hawaiian tropical forests arrayed on diverse soil types associated with geologic substrate age. Moreover, they observed similar gap-size frequency patterns when comparing the floristically unique Hawaiian island forests to a more typical continental tropical forest in the Atlantic lowlands of Costa Rica. The results suggested that, independent of environmental and biogeographic origin, tropical forests may converge on similar size-scaling patterns of canopy gaps. Despite the observation of convergent gap patterns in contrasting Hawaiian and Costa Rican forests, we currently lack the data required to know whether sizes and spatial properties of canopy gaps converge to similar patterns throughout the tropics. Understanding causes of variability in the sizes and spatial properties of canopy gaps is key to modeling ecosystem dynamics, and for estimating the role of abiotic (e.g. soils, climate, topography) and biotic (e.g. floristic composition) factors mediating canopy turnover [4], [14]–[17].

In forests of lowland Amazonia, the sizes and frequencies of canopy gaps may yet be related to environmental factors that vary at multiple spatial scales. Since forest biomass, structure and floristic composition are well known to vary with topography, geology and soils [18]–[22], we might expect canopy disturbance regimes to vary on a similar basis. At the broadest regional scales, geologic substrate imparts a strong set of controls over structure and function [20], [23]. Somewhat independent of geologic origin, many landscapes within the western Amazon region can be partitioned into two very broadly defined forest types: (i) erosional *terra firme* (ETF) forests on elevated terraces with clayey soils often classified as Ultisols to Oxisols; and (ii) depositional floodplain (DFP) forests in low-lying areas near rivers and streams with loamy-to-sandy soils often classified as Inceptisols [24]. Forests on floodplains also often experience seasonal inundation, which deposits sediments from upstream and from neighboring *terra firme*. Combined, these nested abiotic factors could prove important in mediating variation in disturbance regimes as expressed in gap-size frequency distributions.

Characteristics of size-frequency distributions of canopy gaps can be used to quantitatively describe the disturbance regime of a forested landscape. When plotted on a log-log scale, the negative slope of the relationship between gap sizes and their frequency is the exponent (**λ**) of a power-law distribution [7]. As a result, **λ** provides a quantitative measure of the prevailing gap size-frequency pattern in a single parameter, and is thus useful for comparing forests [12], [25]. Previous work has suggested that **λ** values typically range from about 1.0–3.0 in forests, with a threshold value of 2.0 providing a cutoff for whether a forest is dominated by small or large gaps [26], [27].

Using airborne LiDAR, we quantified canopy gap-size frequency distributions throughout the southern Peruvian Amazon basin. Our goal was to assess whether a regional mosaic of geologic, topographic and canopy physiognomic variation imparts differences in forest disturbance regimes as indicated by **λ**-values. Large values of **λ** (>2.0) would suggest a forest dominated by smaller gaps that may be indicative of high growth-low mortality dynamics. Small **λ**-values (<2.0) would indicate the prevalence of larger canopy gaps associated with mortality of large canopy or emergent trees or alterations to whole stands. We ask: Do different lowland forests situated on contrasting terrains and geologies in the Peruvian Amazon harbor intrinsically different gap-size frequency distributions?

## Materials and Methods

### Study Region

**Figure 1:**
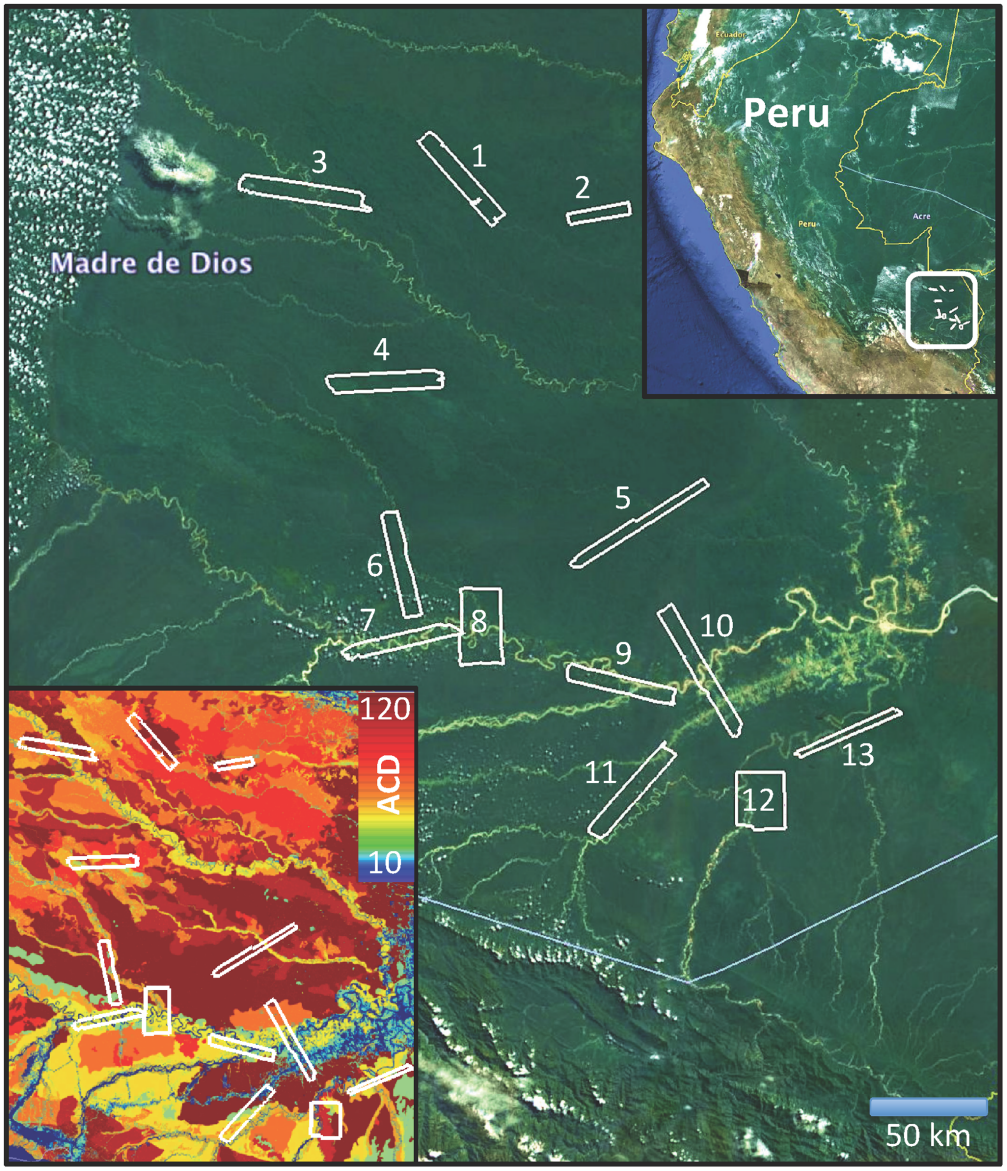
Thirteen CAO LiDAR mapping blocks were acquired in the southern Peruvian Amazon. The upper inset shows location of the study region within Peru. The lower inset shows the LiDAR mapping blocks against a map of aboveground carbon density (ACD; Mg C ha^−1^), which integrates regional variation in geology, topography and canopy physiognomy [20].

The study was undertaken in the Madre de Dios watershed in Peru, throughout a region of well-known geologic, topographic and physiognomic variation stretching from the base of the Andes to the border with the Brazilian State of Acre (Figure 1). Our landscape-scale samples covered forests representing no less than 5 million ha common to the region as a whole [20]. The region contains variable geology and topography [28]. The northern section is dominated by highly dissected, rolling upland terrain dating as far back as the Paleozoic. This region contains few large rivers, yet numerous smaller depositional areas intermingled with the higher *terra firme* [29]. The southern portion of the region can be partitioned into predominantly two landforms: (i) ETF incised with smaller streams of Pleistocene origin; and (ii) large low-lying DFP ecosystems of Holocene origin. Floristic composition and aboveground carbon stocks co-vary with these spatially nested patterns of geology and topography [20]. The region also contains some forests containing mid-story bamboo (“pacal”) associated with the geologic feature known as the Fitzcarrald Arch [30]. This region of the Amazon basin has relatively high soil fertility and rapid tree turnover in comparison to forests in central Brazil and to the northeast on the Guyana Shield [21].

### Airborne LiDAR Data Acquisition

In July 2009, we flew the Carnegie Airborne Observatory (CAO) Alpha system [31] to map 125,581 ha of forest in 13 survey blocks along our regional transect, with each block ranging in area from 225 to 14,062 ha (Table 1). CAO-Alpha included a waveform LiDAR capable of mapping the forest canopy 3-D structure as well as a high-fidelity imaging spectrometer for interpretation and confirmation of forest types throughout the study [20].

**Table 1:** Thirteen airborne LiDAR study blocks were used to map depositional-floodplain (DFP) substrates (68, 958 ha) and a variety of erosional *terra firme* (ETF) substrates (56,623 ha).

Flight	DFP	ETF	ETF
Block	Substrates (ha)	Substrates (ha)	Geologic-Topographic-Physiognomic Descriptor
1	1,573	10,909	Neogene – Low Rolling Hills – Dense Tree Canopy
2	605	2,526	Neogene – Low Rolling Hills – Dense Tree Canopy
3	6,654	6,586	Paleozoic – High Rolling Hills – Dense Tree Canopy
4	–	13,215	Paleozoic – High Rolling Hills – Bamboo-dominated Canopy
5	1,585	8,209	Pleistocene – High Flat Terraces – Mixed Swamp/Tree Canopy
6	4,753	4,133	Pleistocene – High Flat Terraces – Dense Tree Canopy
7	7,826	813	Pleistocene – Low Flat Terraces – Open Tree Canopy
8	14,062	4,726	Pleistocene – Med. Flat Terraces – Dense Tree Canopy
9	8,650	306	Pleistocene – Low Flat Terraces – Dense Tree Canopy
10	225	2,807	Pleistocene – Low Flat Terraces – Dense Tree Canopy
11	8,092	–	–
12	11,300	1,583	Pleistocene – Low Flat Terraces – Mixed Swamp/Tree Canopy
13	3,473	810	Pleistocene – Low Flat Terraces – Mixed Swamp/Tree Canopy
**TOTAL**	**68,958 ha**	**56,623 ha**	

The *terra firme* zones are described in terms of basic geologic, topographic and physiognomic composition.

The CAO flights were conducted at 2000 m above ground level at a ground speed of ≤95 knots. The LiDAR was operated with a 38-degree field of view and 50 kHz pulse repetition frequency, resulting in 1.1 m laser spot spacing. Due to the custom-designed laser beam divergence of the CAO Alpha system (0.56 mrad), each laser shot overlapped by 50% for a continuous LiDAR coverage. In addition, the LiDAR data were collected in parallel flight lines overlapping by 50%, which provided laser point densities averaging 2 pulses m^−2^. Results reported here are for LiDAR measurements digitized in up to 4 discrete returns per laser pulse. The dataset thus contains approximately 10 billion laser returns over the 125,581 ha needed for 3-D analyses of canopy gaps. LiDAR spatial error was previously determined to be <0.15 m vertically and <0.36 m horizontally (RMSE) [20], [32].

### LiDAR Data Processing

Laser ranges from the LiDAR were combined with the embedded Global Positioning System-Inertial Measurement Unit (GPS-IMU) data [31] to determine the 3-D locations of laser returns, producing a ‘cloud’ of LiDAR data. The LiDAR data cloud consists of a very large number of georeferenced point elevation estimates (m), where elevation is relative to a reference ellipsoid (e.g., WGS 1984). To estimate canopy height above ground, LiDAR data points were processed to identify which laser pulses penetrated the canopy volume and reached the ground surface. We used these points to interpolate a raster digital terrain model (DTM) for the ground surface. A 30 m x 30 m kernel was passed over each flight block and the lowest elevation estimate in each kernel was assumed to be ground. Subsequent points were evaluated by fitting a horizontal plane to each of the ground seed points. If the closest unclassified point was <5.5 degrees and <1.5 m higher in elevation, it was classified as ‘ground’. This process was repeated until all points within the block were evaluated. The DSM was based on interpolations of all first-return points (i.e. it includes canopy top and ground if ground was the first return, which would indicate bare ground surface exposed to the sky, such as a 0 m gap). Measurement of the vertical difference between the DTM and DSM yields a model of canopy height above ground (digital canopy model, DCM).

### Forest and Terrain Classification

To compare gap-size frequency distributions among forests in the lowland Peruvian Amazon, we classified each LiDAR block by its geologic, topographic and physiognomic composition (Table 1, Figure 1). The Peruvian government’s geologic [29] and vegetation maps were combined with NASA Shuttle Radar Topography (SRTM) data to develop a basic classification of the erosional *terra firme* portions of each study block as reported in Asner et al. [20]. In contrast, depositional areas including floodplains receive minerals, soil, organic matter and nutrient inputs from multiple sources nested throughout river-catchment networks. As a result, all floodplains and stream systems are classified here as one type of Holocene origin (Table 1).

To partition each study block into depositional-floodplain (DFP) and erosional *terra firme* (ETF) sectors, the LiDAR DTM was used to model height above nearest river (or stream). A threshold value in height above nearest river was then determined iteratively to best delineate these two contrasting surfaces. We found that a threshold of 15 m best separated them, which we confirmed with field checks in study blocks 8 and 12.

### Gap-size Frequency Analysis

We defined gaps in the forest canopy by applying a definition similar to Brokaw’s [33] definition to the DCM results. In the classical sense, gaps are openings in the forest canopy extending down to an average height ≤2 m aboveground [33]. However, because canopy height variations are continuously distributed, we quantified the number and sizes of openings in the forest canopy in 1-m vertical slices [13]. This extension of Brokaw’s definition was thus applied to a range of gap-depth classes, permitting analysis of all gaps extending from the top of the canopy to different heights aboveground.

**Figure 2:**
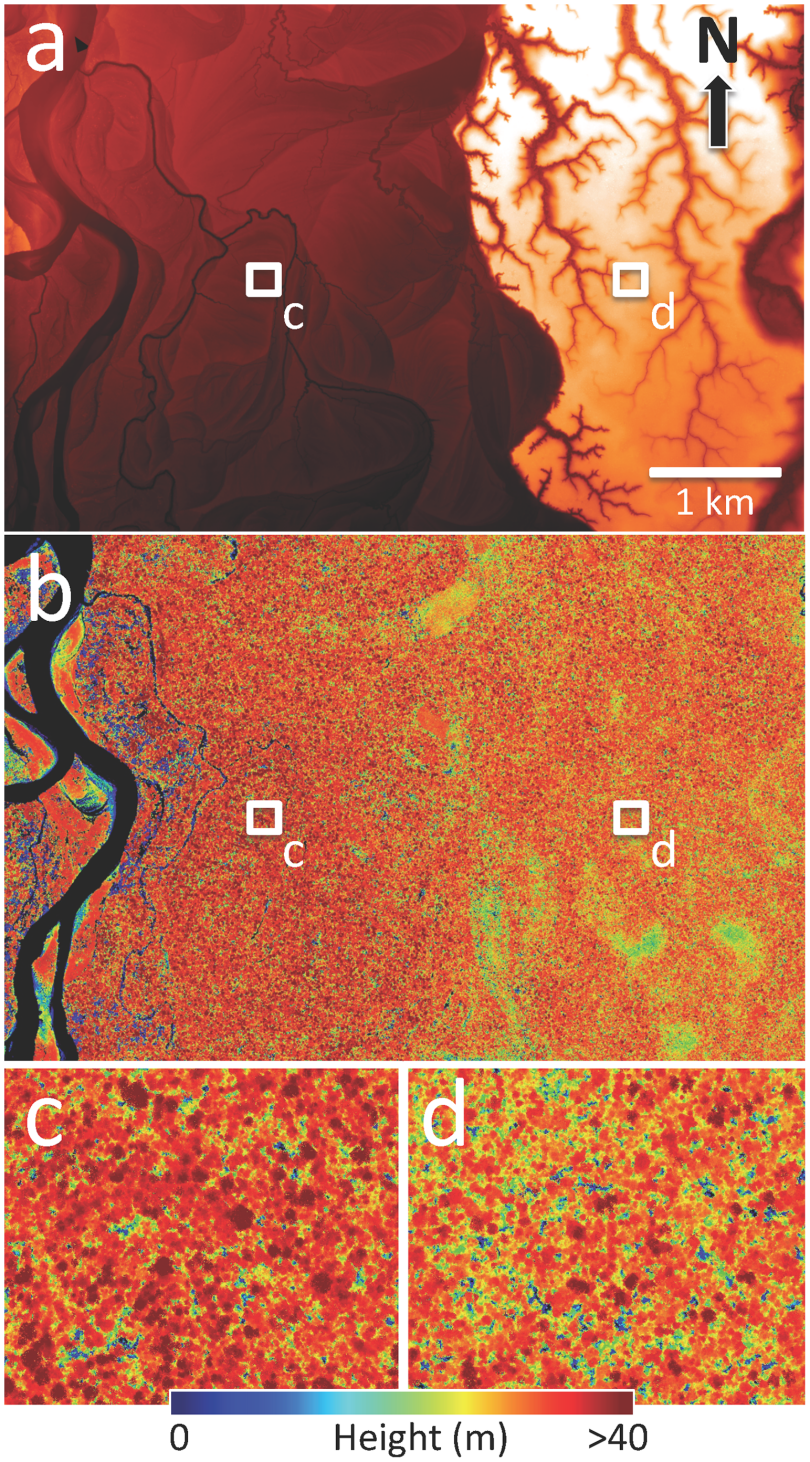
One 13,883 ha Amazonian landscape (block 12;Figure 1) showing (a) the digital terrain model with additional processing to delineate depositional floodplain (DFP; red) and erosional *terra firme* (ETF; white) substrates; (b) forest canopy height derived from 3-D imaging; and zoom images to indicate differences in height and gap variation within (c) DFP and (d) ETF forests. Each zoom image is 50 ha in size. Individual crowns are visible in red colors; forest canopy gaps are indicated in blue.

Because LiDAR-based analyses yield gap data at all heights aboveground, we initially focused attention in one of our core study landscapes (block 12, Figure 2) to seek ways of reducing the data volume to a few meaningful thresholds at which to report gap size-frequency results. We selected block 12 because this area has been a focus of very extensive ground-based research on canopy composition, structure, function and remote sensing [34]–[36]. Through the analyses to be presented in the results section, we found that two gap thresholds (≤1 m and ≤20 m) were sufficient to represent the overall pattern of static gaps on these two landscapes. Gaps associated with the ≤1 m threshold can be thought of as whole-tree and large canopy branch failures; those with the ≤20 m threshold can be considered as failures of crowns and branches mostly in the upper canopy. We applied these 1-m and 20-m thresholds to all blocks throughout the study, facilitating comparisons of gap-size frequency at block (i.e., landscape) and regional scales.

To quantify the size frequency distribution of canopy gaps at 1-m and 20-m height thresholds, we used the Zeta distribution, which is a discrete power-law probability density. For the Zeta distribution with parameter **λ**, the probability that gap size takes the integer value *k* is:
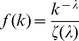
(1)where the denominator is the Riemann zeta function, and is undefined for **λ**  = 1. A detailed narrative and syntax for carrying out this procedure using R is provided in Appendix S1 [37]. This distribution is sometimes called the ‘discrete Pareto distribution’, and is appropriate for modeling the size-frequency of canopy gaps [27], [38], [39]. We calculated maximum likelihood estimates (MLE) of **λ** by minimizing a negative log-likelihood function [39].

## Results

### Block 12

We found that the core study area of block 12 was representative of three regional patterns describing forest structure and size-frequency distributions of canopy gaps. These patterns were consistent across all blocks: (i) significant differences in canopy height between DFP and ETF substrates; (ii) similarity in scaling exponents of gap size-frequency distributions despite height differences on adjacent substrates, and (iii) often more gaps in ETF forests than in DFP forests. For additional reference, we provide the gap-size frequency distributions, MLEs, and sample sizes for all forest blocks in Appendix S2.

**Figure 3:**
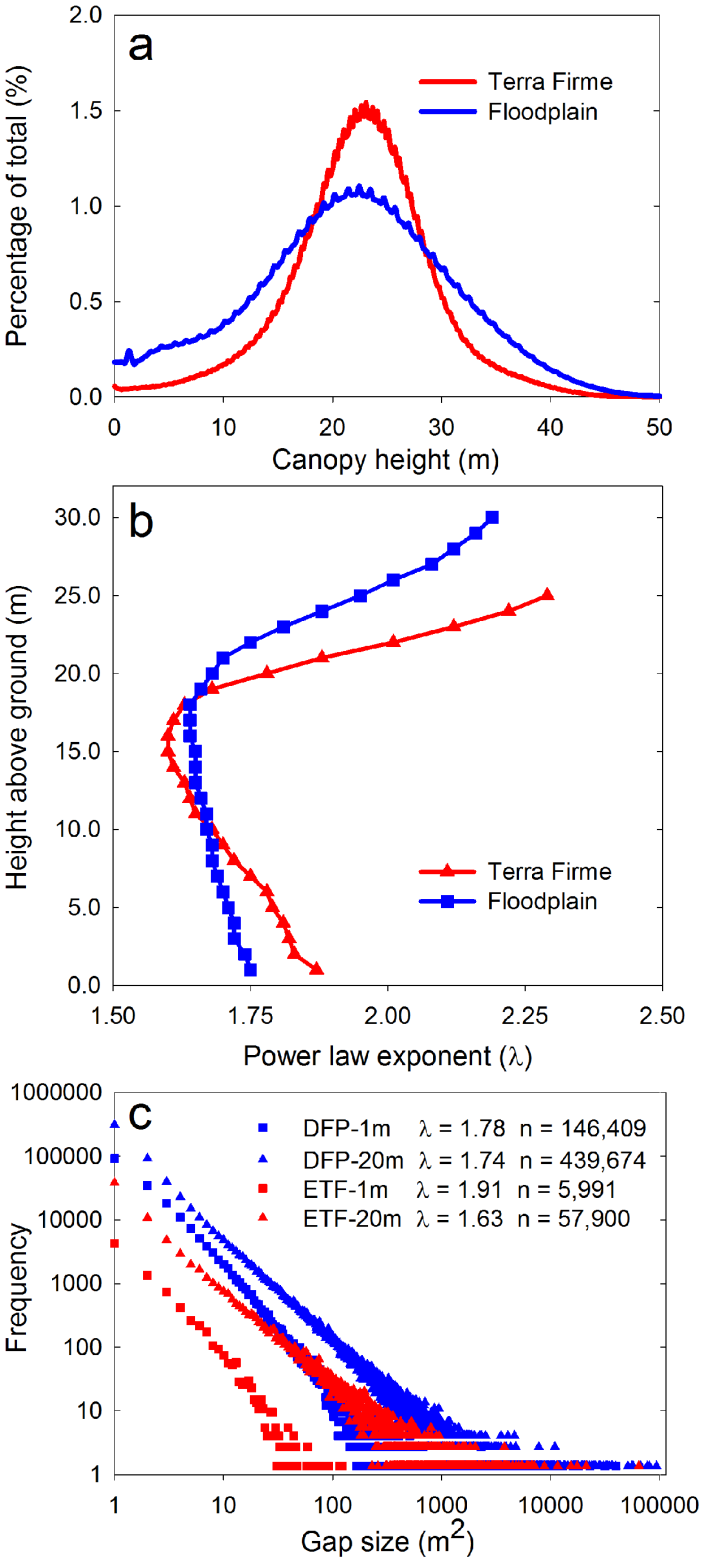
Canopy structure and gap statistics for CAO mapping block 12 including: (a) the distribution of canopy height for erosional *terra firme* (ETF) and depositional floodplain (DFP) forests (see Figure 2); (b) the vertical distribution of power-law exponents (λ) for each forest type in block 12; and (c) the gap-size frequency distributions for ETF and DFP forests for canopy gaps at <1 m and <20 m thresholds. Power-law exponents (λ) and the number of mapped gaps (n) are also provided.

In block 12, the mean (± s.d.) height of DFP forests (20.2±10.5 m) was slightly but significantly lower than ETF forests (22.5±6.5 m) (t-test; *p*<0.05) (Figure 3a). However, there was a much wider range of height values in the DFP compared to the ETF, and much taller trees could be found throughout the DFP (Figure 2b,c). Despite these differences in the mean and variance of canopy height, the vertical distribution of **λ**-values in the forest canopies was similarly shaped in each forest type (Figure 3b). Both showed a hump-shaped distribution of **λ** that peaked at intermediate height classes. Smaller values (**λ**  = 1.60–1.85) occurred from ground level to about 20 m, above which **λ** increased. The absolute minima were reached at 16 m and 18 m in ETF and DFP forests, respectively. Thereafter, **λ** increased to about 25 and 30 m in each forest type, respectively.

Uniformity of the vertical distributions of **λ** (Figure 3b), even with contrasting height distributions (Figure 3a), indicated that we could compress the reported data volume into two integrated vertical height classes. The first class includes all gaps with vegetation ≤1 m in height. The second class includes all gaps with vegetation ≤20 m in height. In this latter case, we are considering the gap-size distribution for only the smaller gaps found in the mid-to-upper canopy.

We next show the size-frequency distributions in block 12 for canopy gaps with vegetation ≤1 m and ≤20 m in height (Figure 3c). In both DFP and ETF forests, gap-size frequency followed the power-law Zeta distribution. In the case of block 12, gap numbers were larger in the 20-m class than in the 1-m class, on both DFP and ETF substrates. However, we also found that the total number of gaps was much higher on DFP than on the ETF at this site (Figure 3c). The parameter **λ** was similar for gaps ≤1 m and ≤20 m in height for DFP forests. Based on the findings for block 12, we compared canopy height distributions and **λ**-values at the 1-m and 20-m vegetation height thresholds for the remaining blocks (Appendix S2).

### Canopy Height Distributions

Mean (± s.d.) canopy heights ranged from minimum of 12.1±11.5 m to a maximum of 24.9±7.8 m across all study blocks (Table 2). Regionally, ETF forests had canopies with heights that were 17% taller than those on DFP. However, in any given landscape or sampling block, ETF were 4–82% taller than their paired DFP sites. In 9 of 13 blocks, the height ranges were substantially larger in the DFP. There were no significant relationships between mean canopy height and **λ** with the exception of the DFP 1-m gap class (*r*  = 0.81, *p*<0.01).

**Table 2:** Mean canopy height (± standard deviation) of forests on depositional-floodplain (DFP) and erosional *terra firme* (ETF) substrates (see Table 1), along with Zeta distribution (power-law) exponents (**λ**) of the gap-size frequency distributions for each site.

Block	DFP Substrates			ETF Substrates		
	Height (SD)	λ1	λ20	Height (SD)	λ1	λ20
1	22.5 (9.3)	1.93 (9020)	1.81 (71751)	23.6 (8.8)	1.95 (44803)	1.87 (614784)
2	21.8 (8.3)	1.93 (2755)	1.96 (29367)	22.9 (8.5)	2.00 (8979)	1.84 (115469)
3	21.6 (9.6)	1.95 (43144)	1.90 (311181)	24.8 (9.2)	1.98 (20491)	1.80 (347253)
4	–	–	–	22.5 (10.7)	1.73 (175825)	1.76 (613018)
5	23.9 (8.7)	1.97 (9137)	1.83 (91764)	24.9 (7.8)	1.93 (38004)	1.72 (450834)
6	20.6 (8.8)	1.81 (36329)	1.81 (178283)	23.4 (8.1)	1.82 (24669)	1.68 (160665)
7	12.1 (11.5)	1.70 (331647)	1.87 (307547)	22.0 (8.4)	1.75 (9238)	1.72 (33536)
8	19.0 (9.8)	1.89 (150971)	1.86 (483723)	23.7 (8.4)	1.85 (24212)	1.65 (173344)
9	15.4 (11.9)	1.80 (138192)	1.92 (271617)	21.6 (8.4)	1.85 (1461)	1.74 (11661)
10	18.0 (7.5)	1.71 (4916)	2.03 (3317)	22.3 (7.5)	1.74 (44851)	1.85 (35987)
11	20.3 (7.2)	1.88 (28985)	1.72 (261803)	–	–	–
12	20.2 (10.5)	1.78 (146413)	1.74 (439677)	22.5 (6.5)	1.91 (5995)	1.63 (57901)
13	19.5 (10.3)	1.80 (38208)	1.75 (122226)	21.0 (6.4)	1.88 (3343)	1.74 (26605)

Values are provided for gaps reaching to the ground level (**λ1** or ≥1 m) and for gaps found only in the upper canopy (**λ**20 or ≥20 m). Values in parentheses indicate the number of gaps mapped in each landscape.

### Gap-size Frequency Distributions

We mapped 1,000,703 gaps with vegetation ≤1 m in height (Table 2). Averaged across blocks, the **λ**-values for these gaps were 1.86 (±0.08) and 1.88 (±0.09) on DFP and ETF substrates, respectively. Within blocks, **λ**-values were also very similar on DFP and ETF surfaces, with the largest difference of just 7% found between the two substrates in block 12. Regionally, **λ**-values were 8–9% higher, indicating smaller gaps overall, in DFP areas on the much older, rolling terrains in the north (blocks 1–4) as compared to southern blocks containing wider floodplains (blocks 9–13). A similar yet even weaker pattern was observed on ETF, where **λ**-values were approximately 4% higher in the more dissected northern blocks as compared to the southern flat terraces of Pleistocene origin (Table 2).

We mapped 4,877,234 gaps with vegetation height ≤20 m (Table 2). Averaged across blocks, the **λ**-values for these gaps were 1.85 (±0.10) and 1.75 (±0.08) on DFP and ETF substrates, respectively. Again, the **λ**-values were 3–7% higher in the northern blocks with more narrow floodplains and more dissected erosional surfaces, but the pattern was even less predictable for many of the individual landscapes (Table 2). Overall, the range of **λ**-values, whether representing gaps with vegetation height ≤1 m or 20 m, was small across all landscapes, and on both DFP and ETF substrates.

## Discussion

### Similar Gap-size Frequency Distributions

**Figure 4:**
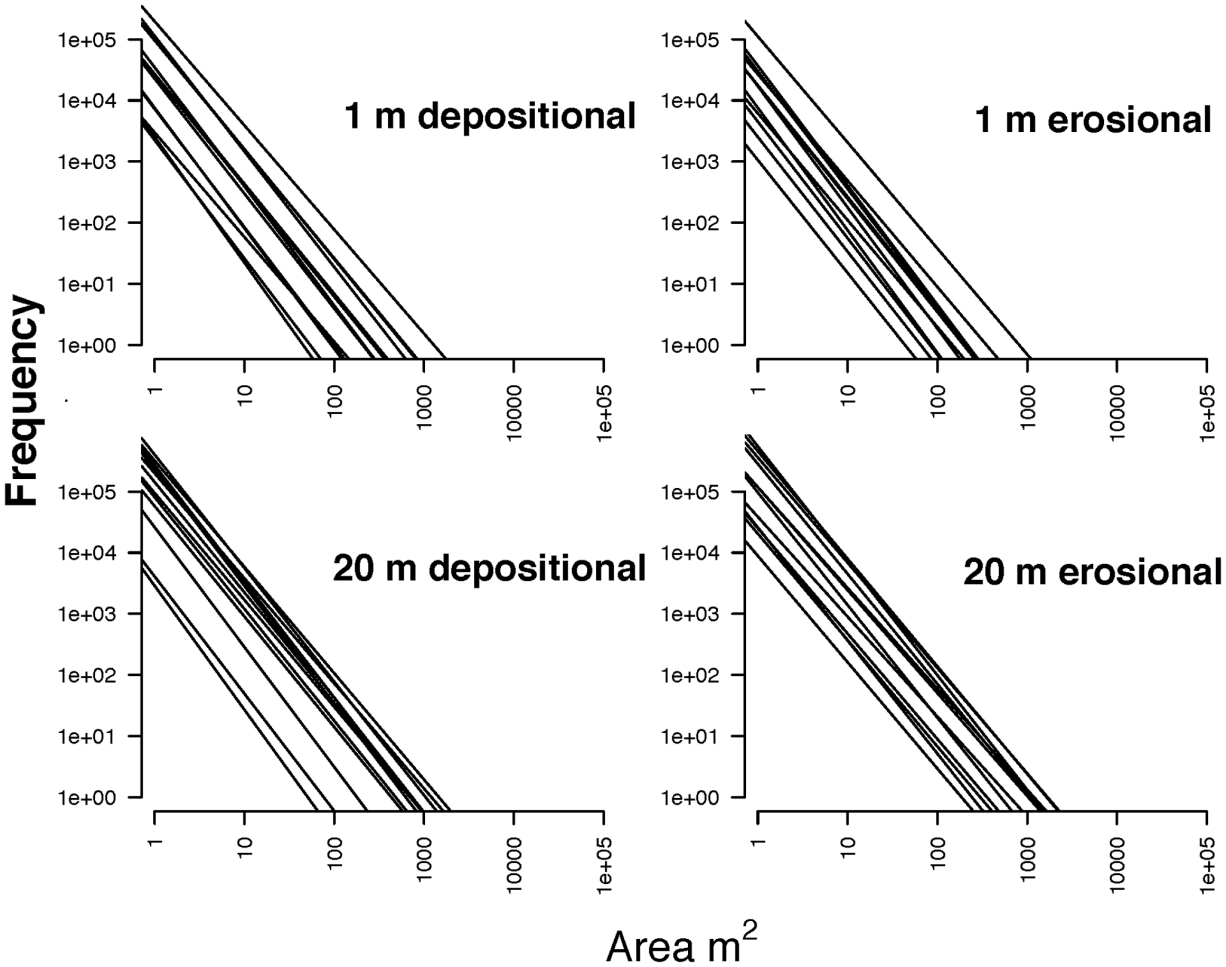
Graphical representation of size-frequency distributions of canopy gaps on erosional *terra* firme and depositional floodplain substrates in the southwestern Peruvian Amazon at two height thresholds. The slopes of these lines are power-law exponents from the Zeta distribution that we estimated using maximum likelihood. Additional details are in Appendices S1 and S2.

Size frequency distributions of canopy gaps were largely invariant among forests on erosional-*terra firme* and depositional-floodplain substrates in 125,581 ha of the Peruvian Amazon basin. Scaling exponents varied <7.0% across wide-ranging geologic, topographic, and physiognomic conditions typical of southwestern lowland Amazon forests, as indicated in a summarized comparison of the size-frequency distributions in Figure 4. This surprising degree of similarity in gap-size frequency distributions indicates convergent structural responses to canopy failure, which are independent of regional- and landscape-scale variation in soil fertility, hydrological conditions, and a host of other factors. This occurs despite the fact that canopy height varies within landscapes and regionally (Table 2). Moreover, because aboveground carbon stocks are tightly linked to tree height [20], [40], [41], it follows that regional patterns of carbon storage will be unrelated to size-frequency distributions of canopy gaps.

The repeatability of the power-law relationship for representing gap-size frequency distributions, combined with the observed limited range of values in its scaling coefficient **λ**, suggests that lowland Amazonian forest canopies display similar gap-scaling processes across a wide range of floristic and environmental conditions. This may, in turn, be traced to similarity in how trees fill three-dimensional space in mature tropical forests. Much theoretical work has focused on providing an explanatory foundation for understanding canopy space-filling patterns, particularly using metabolic scaling theory and cellular automata [42], [43]. If our observations of consistent gap-size frequency patterns are indeed tied to canopy space-filling patterns, then LiDAR-based surveys will provide a useful constraint over models of canopy space-filling processes. Independent of whether one works with gaps or with filled space in the canopy, no study has definitively explained the biological or ecological causes for such a high level of scaling consistency. General thinking on the matter has long invoked resource limitation – particularly light – as a driver of the consistent space-filling patterns we observe in many forests [44], [45]. Our large-scale, high-resolution observations could be combined with existing models to advance our understanding of the evolution of tropical forest canopy structure and architecture.

### Southwestern Amazonian Disturbance

Amazonia has emerged as an epicenter for canopy analysis of forest disturbance due to disagreement about the role of the Basin in the carbon cycle [26], [27], [46]–[48]. Studies using time series satellite observations suggest that mesoscale weather-related disturbances, referred to as blowdowns, reset the forest successional clock by severely damaging large stands of trees [49]–[51]. While there is no doubt that such large-scale disturbances initiate secondary succession, the debate rests on whether these events are geographically widespread across the Basin, as well as how frequently they occur in any location. Meanwhile, plot-scale studies suggest that forest carbon accumulation is increasing through time [52], [53], not as recovery from large-scale blowdowns, but perhaps as a response to atmospheric CO_2_ fertilization enhancing growth over mortality [54].

Although our sampling of 125,581 ha of forest, representing differing abiotic and biotic conditions found throughout millions of hectares of the southwestern Amazon, is the largest-scale, highest-resolution mapping of canopy gaps to date, it cannot account for the possibility that a once-per-century blowdown might have reset the carbon accumulation clock somewhere within the study region. With blowdowns occurring so infrequently, the LiDAR mapping would need to cover even more geography to detect such large-scale disturbances [48]. And while we did not detect the presence of blowdowns in this study, a similar style of LiDAR sampling did reveal one ∼2,000 ha blowdown in 465,000 ha of forest sampled in the Colombian Amazon [55]. The inclusion of large-scale, extremely infrequent gap forming events thus remains a hit-or-miss undertaking when using airborne techniques. Integration with wall-to-wall satellite mapping approaches are needed to determine their contribution [56].

Notwithstanding extremely rare disturbance events, the wide spatial coverage and high resolution of the LiDAR measurements provide a uniquely robust sampling of landscape-scale disturbance regimes in southwestern Amazonia. Nearly all of our landscape **λ**-values were 10–20% lower than 2.0, which is the threshold used to define forests subjected to larger disturbances versus those undergoing much smaller, finely-grained dynamics [26], [27]. Although Fisher et al. [27] suggested that Amazonian forests have intrinsic **λ**-values in the 1.1–1.6 range, which would indicate the prevalence of massive, stand-resetting disturbances, Lloyd et al. [26] later recalculated their results to produce an estimated **λ** range of 1.9 to 3.1. Our results strongly suggest that southern lowland Peruvian forests are at the lower end of this range, and thus these forests are subject to relatively large gap-forming processes, likely associated with large crown turnover as well as the prevalence of fairly strong winds and storms known regionally as “friaje” [57].

Beyond the comparisons to Fisher et al. and Lloyd et al., additional comparisons of our gap-size distributions to other tropical forests remain limited at this time, owing to the scarcity of tropical studies in the literature. Kellner et al. [13] reported **λ**-values of 1.99 and 1.66 for canopy gaps with vegetation height ≤1 m and ≤20 m aboveground for a lowland Costa Rican tropical forest. In comparison to their site, we found southwestern Amazonian forests to harbor larger gaps on average that extend from top-of-canopy down nearly to the ground (**λ**  = 1.87 for ≤1 m gaps), but relatively smaller gaps in the upper canopy (**λ**  = 1.80 for ≤20 m gaps). However, a variety of submontane Hawaiian forests contained stands with **λ**-values ranging from 1.8 to 2.6, even though the stands were each dominated by a single keystone Hawaiian canopy species, *Metrosideros polymorpha* [12]. This suggests that gap-size frequency exponents can vary substantially even in the absence of floristic compositional changes, and very much in response to abiotic factors including soils and terrain. Our results, however, indicate quite the opposite – that widely varying environmental conditions (albeit all in lowland Amazonia) do not impart an identifiable pattern in the spatial scaling of forest canopy gaps.

We note that our results were fairly consistent at both the 1-m and 20-m vegetation height thresholds (Table 2). The **λ**-values were an average 3% lower in the 20-m than in the 1-m class for the majority of floodplain and terra firme landscapes, which may indicate a slightly elevated degree of larger gaps in the upper canopy. However, the pattern was extremely variable, with **λ** up to 15% lower in the 20-m than in the 1-m class on *terra firme* in our core site – block 12. Moreover, block 10 contained a wide floodplain harboring canopy gaps that were 20% larger in the 20-m class as compared to the 1-m class. Reviewing the canopy height maps among all sampling blocks, we think that variation in gap-size distributions in floodplain environments is more an expression of hydrologically-mediated disturbance (e.g., seasonal flooding) than it is of an underlying floristically-based process affecting gap-size frequencies.

Independent of the vegetation-height thresholds used in this study, we see a need for standardization in the measurement and reporting of forest canopy gap distributions using LiDAR. This technology can image a forest in 3-D at resolutions ranging from meters to centimeters, resulting in different gap-size detections based simply on spatial resolution. Moreover, the data can be partitioned by vertical stratum in the canopy, as we showed in Figure 3b, leading to variation in gap-size frequency estimates vertically that may exceed those derived among comparative forests (i.e., see **λ**-values in Figure 3b vs. Table 2). Here we presented results from LiDAR measurements made with an average of two pulses m^−2^ and a laser beam divergence that preserves full overlap between adjacent laser spots to provide continuous spatial coverage. Other LiDAR systems, measurement specifications, and analytical approaches will result in different gap-size frequency distributions, and thus potentially different estimated values of **λ**. We suggest that, in the minimum, future reports provide specific information on the LiDAR measurement settings, pixel size, flight parameters, instrumentation and the portion of the vertical canopy profile of interest. Better yet, we further suggest a nominal sampling protocol: Through extensive testing, we have found that ∼1 m spot spacing with ≥2 pulses m^−2^ provides a detailed set of canopy measurements that can be achieved with nearly any airborne scanning LiDAR system in operation today.

### Limitations

Despite our relatively straightforward results, we also recognize several limitations in the study. First, we are only reporting on the southern Peruvian lowland Amazon. The results presented here need to be compared to forests in the foothills and montane transition from the lowlands up into the Andes, as well as in other lowland forests throughout Amazonia. Given that forest carbon stocks, productivity and floristic composition vary widely across the Basin [21], [58], our results should not be used to represent the entire region as a whole. Second, we present gap-size frequency distributions for large landscapes that average local-scale variation in floristic composition, such as swamp-dominated vegetation, palm forests, and pockets of bamboo forest, all of which occurs in smaller fractions of the landscapes we reported. These localized variations in forest composition are likely to impart variation in gap-size frequency distributions, but the magnitude of their impact on our conclusions is not known.

Like most LiDAR-based studies, this work imaged static gaps in the canopy. A static gap is an opening in a forest canopy at a given point in time. When such an opening was formed is unknown at the time of observation, and thus static gaps confound time with disturbance intensity (i.e. square meters of canopy loss). As such, it remains unknown whether any given gap is shrinking due to regeneration or growing due to adjacency effects from neighboring individuals [59], [60]. The best way around this problem is to image canopy disturbances directly to derive recent disturbance events. Our forthcoming reports will include results from repeat mapping to derive dynamic rates and patterns of tropical forest gap formation and closure.

## Supporting Information

Appendix S1 (DOCX)

Appendix S2 (DOCX)
