# Supplementary material for: Forest Canopy Gap Distributions in the Southern Peruvian Amazon
Source: PLoS One. 2013 Apr 15;8(4):e60875. doi: 10.1371/journal.pone.0060875 (PMC3626694; doi:10.1371/journal.pone.0060875)
Supplement: Appendix S1 — (DOCX) [file pone.0060875.s001.docx]

**Appendix S1: Statistical computation of size-frequency distributions of canopy gaps using remote sensing data.**

from ***Forest Canopy Gap Distributions in the Southern Peruvian Amazon***

Gregory P. Asner, James R. Kellner, Ty Kennedy-Bowdoin, David E. Knapp, Christopher Anderson, and Roberta E. Martin

This tutorial describes a framework for analysis and visualization of size-frequency distributions of canopy gaps using remote sensing data. We first describe image processing methods to distinguish canopy gaps, and then proceed to analysis and visualization using the R program and environment for statistical computation [1].

We define canopy gaps as contiguous areas where vegetation height is ≤ some height threshold aboveground. Typically this threshold is 2 m [2], but in practice the gap versus non-gap dichotomy is overly simplistic, because openings in forest canopies expose vegetation of a wide range of heights above ground [3,4]. For example, in some cases the death of a canopy tree may expose sub-canopy vegetation that is 5 – 10 m in height. The particular value for a height threshold is not important to this tutorial. Our algorithm proceeds in the following steps:

1. Identify a pixel that is < the minimum-height threshold. Assign this pixel a unique label.
2. Evaluate the 8 neighbors of the pixel identified in (1). If they are < the minimum-height threshold, and therefore part of the same canopy gap, assign them the same label.
3. Repeat (2) until there are no neighbors < the minimum-height threshold. This completes the identification of a single canopy gap.
4. Repeat (1) – (3) until there are no remaining unlabeled pixels in the image.
5. Count the number of pixels within each uniquely-labeled canopy gap. These numbers provide the basic information required to visualize and analyze the size-frequency distribution.

The size-frequency distribution is a summary that counts the number of times that gaps of a given size are represented in data (e.g., the number of gaps that are 1-pixel in area, 2-pixels in area, etc.). We analyze gap size in terms of numbers of pixels because remote sensing data in the form of images are fundamentally discrete. Even though LiDAR height measurements are continuous measurements, the estimates of gap sizes they distinguish are not. To see this, consider the sizes of gaps that are possible when pixel-size has 1.1 m side length. The smallest possible gap is a single pixel, which would be 1.21 m^2^. The next smallest would be a two-pixel gap of 2.42 m^2^. Note that it is impossible to see a gap of exactly 2 m^2^ using these data. Thus, continuous distributions (e.g., Pareto) should not be used [5]. The Zeta distribution (also sometimes called the discrete Pareto distribution) is a discrete power-law distribution. It predicts integers > 0 with size-frequencies that are power-law distributed. These distributions have a negative-slope when size-frequencies are plotted on logarithmic axes, and can be described using the scaling parameter *λ*. This parameter, sometimes also called the ‘scaling exponent’, or simply ‘exponent’, takes on positive values > 1. Larger values of *λ* correspond to steeper size-frequency distributions (i.e. fewer large events [3]).

We have written R functions to (1) simulate data under a Zeta distribution, (2) calculate the probability density of data under a Zeta distribution, given a value for the scaling exponent and (3) calculate the negative log-likelihood of data under a Zeta distribution, given a value for the scaling exponent. We describe how to use each of these functions to generate maximum likelihood estimates of *λ* below. We have included syntax that can be entered directly into R. R syntax is distinguished from plain text using Courier font. We begin with three functions.

# Random generation for the Zeta distribution with parameter lambda. n is the # number of observations that will be generated. max is the maximum allowable # value that can be observed, and should be set to a very large integer. More # on this below.

rdpareto <- function(n, lambda, max){

x <- 1:max;sample(x, size=n, replace=T, prob=ddpareto(x, lambda))

}

# Density function for the discrete Pareto distribution with parameter lambda

ddpareto <- function(x, lambda){

require(VGAM)

x^-lambda/zeta(lambda)

}

# A function to return negative 2 times the log-likelihood of data under a

# discrete Pareto distribution with scaling parameter lambda.

dplik <- function(data, lambda){

2*sum(-log(ddpareto(x=data, lambda=lambda)))

}

To understand how these functions work, after copying and pasting the text above into R, simulate 1000 observations under a Zeta / discrete Pareto distribution with a scaling parameter equal to 2.

simdata <- rdpareto(n=1000, lambda=2, max=1000000)

To visualize the size frequency distribution of the simulated data, we can use the histogram function, which also counts the number of observations within incremental, unit size classes.

hist(simdata, br=seq(0,max(simdata),1))

The argument br=seq(1,max(simdata),1)) tells R that we want the histogram bins to go from 1 to the maximum value observed in simdata in increments of 1. This produces the histogram below.

This histogram is not particularly helpful, because most of the observations are clustered near the small-size end of the X axis, as expected for power-law-distributed data. We can extract the counts of observations within each size class, and then plot them on a logarithmic scale.

thehist <- hist(simdata, br=seq(0,max(simdata),1), plot=F)

plot(y=thehist$counts, x=thehist$breaks[2:length(thehist$breaks)], log="xy", axes=F)

axis(1)

axis(2)

This produces the familiar linear relationship between frequency and size on a log-log plot. We can use the simulated data to prove to ourselves that we can estimate the value of the scaling parameter *λ* using the other functions. Because these are simulated data using a scaling exponent = 2, we know that the estimated value for lambda should be very close to 2. We will use maximum likelihood to estimate the parameter lambda. First, use the function ddpareto to calculate the probability density of the data using a scaling exponent = 1.75 (i.e. the ‘wrong’ value).

thedensity <- ddpareto(x=simdata, lambda=1.75)

Take a look at the first 10 values, and compare them to the first 10 observations in the simulated data.

thedensity[1:10]

simdata[1:10]

This will show probability densities associated with each datum. The product of these densities for all of the data is the likelihood of the data. The text below will demonstrate how to find the value of the scaling exponent that is associated with the maximum value of the likelihood. Because the likelihood of the data will be an extremely small number, convention is to work with logarithms. The sum of the logarithm of the probability densities is equivalent to the logarithm of the product of the unlogged densities. The function dplik, above, returns -2 times the sum of the logged probability densities.

dplik(data=simdata, lambda=1.75)

Using our simulated data, this number is 3304.269. Your number will differ because these data are stochastically simulated. Try a value for lambda that is closer to the truth:

dplik(data=simdata, lambda=1.85)

This returns 3256.557 for my data – a smaller number. This is intuitively reasonable – the negative log-likelihood should become smaller as the scaling exponent becomes closer to the true value, which we know is 2 in this case. To see this, use a loop to plot the likelihood for a range of values of the scaling exponent.

lambdas <- seq(1.5,2.5,0.01)

likelihoods <- matrix(NA, nrow=length(lambdas), ncol=1)

for (i in 1:length(lambdas)){likelihoods[i,1] <- dplik(data=simdata, lambda=lambdas[i])}

plot(likelihoods, x=lambdas)

This figure shows that the likelihood reaches a minimum at a value very close to 2. To extract the value of lambda associated with the minimum value of the likelihood, use:

lambdas[which(likelihoods==min(likelihoods))]

This indicates that the minimum is associated with *λ* = 2.01. This graphical approach is fine for a simulated data set, but dealing with large amounts of data and bootstrapping to calculate standard errors require a faster solution. This can be achieved using R’s built-in optimize function.

fit <- optimize(dplik, data=simdata, lower = 1.0001, upper = 20, maximum = F)

The arguments lower and upper are search bounds that the function will not exceed. The maximum argument tells the function whether to minimize or maximize the function. This returns an object called fit that contains two values. Typing fit$minimum is the value of the scaling exponent that minimizes the negative log-likelihood function (i.e. the MLE), and fit$objective is the value of the negative log-likelihood associated with the MLE. To construct a hypothesis test for the MLE, we can estimate the standard error of the scaling exponent. The most straightforward way to do this is using a non-parametric bootstrap. The steps are: (1) draw n samples from the data with replacement, where n is the number of observations in the data, (2) estimate the MLE for the sampled data, (3) repeat 1 – 2 a large number of times, (4) summarize the distribution of MLEs from the sample data.

boot <- matrix(NA, nrow=1000, ncol=1)

for (i in 1:1000){x <- sample(simdata, size=1000, replace=T)

boot[i] <- optimize(dplik, data=x, lower = 1.0001, upper = 20, maximum = F)$minimum}

hist(boot)

sd(boot)

quantile(boot, c(0.025, 0.975))

There are some caveats to this tutorial. First, when simulating data using the function rdapreto, the value for the argument max is the largest possible observable value. This is required because the method being used to generate these data is inverse-distribution sampling [6]. It works in this case by sampling a vector of integers over the range 1 – max using probability densities calculated using the given value for the scaling exponent. You should perform simulations to determine how large max needs to be to avoid sampling a truncated distribution. For example, if the value of the scaling exponent is 1.2, max = 100 is clearly too small. Here is a histogram of 10,000 observations from a Zeta distribution with a scaling exponent of 1.2, and max = 100)

simdata2 <- rdpareto(n = 10000, lambda = 1.2, max = 100)

hist(simdata2)

This histogram clearly shows that the samples are running up against the maximum allowable value. Put another way, this is telling us that a Zeta distribution with a scaling parameter = 1.2 is predicting the occurrence of values > 100. This means that our sample will not closely approximate a Zeta distribution with a scaling parameter of 1.1. To see this, estimate the MLE.

fit2 <- optimize(dplik, data=simdata2, lower = 1.0001, upper = 20, maximum = F)

fit2

This shows that the MLE is approximately 1.47, which is very different from the value we used to simulate the data (1.2). Now change max to 1,000,000, simulate the data again, and estimate the MLE. This will increase the processing time noticeably.

simdata3 <- rdpareto(n = 10000, lambda = 1.2, max = 1000000)

fit3 <- optimize(dplik, data=simdata3, lower = 1.0001, upper = 20, maximum = F)

fit3

Now the MLE is 1.24, much closer to the true value of 1.2. As a general rule, smaller exponents require a larger value for max to simulate data using these functions.

1. Team RDC (2011) R: a language and environment for statistical computing. Vienna, Austria.

2. Brokaw NVL (1982) The definition of treefall gap and its effect on measures of forest dynamics. Biotropica 14: 158-160.

3. Kellner JR, Asner GP (2009) Convergent structural responses of tropical forests to diverse disturbance regimes. Ecology Letters 12: 887-897.

4. Kellner JR, Clark DB, Hubbell SP (2009) Pervasive canopy dynamics produce short-term stability in a tropical rain forest landscape. Ecology Letters 12: 155-164.

5. White EP, Enquist BJ, Green JL (2008) On estimating the exponent of power-law frequency distributions. Ecology 89: 905-912.

6. Clark JS (2007) Models for Ecological Data. Princeton, NJ: Princeton University Press.
