# Supplementary material for: Forest Canopy Gap Distributions in the Southern Peruvian Amazon
Source: PLoS One. 2013 Apr 15;8(4):e60875. doi: 10.1371/journal.pone.0060875 (PMC3626694; doi:10.1371/journal.pone.0060875)
Supplement: Appendix S2 — (DOCX) [file pone.0060875.s002.docx]

**Appendix S2: Size-frequency distributions of canopy gaps from 13 flight blocks in the southwestern Peruvian Amazon.**

from ***Forest Canopy Gap Distributions in the Southern Peruvian Amazon***

Gregory P. Asner, James R. Kellner, Ty Kennedy-Bowdoin, David E. Knapp, Christopher Anderson, and Roberta E. Martin

Each figure shows the size-frequency distribution on depositional flood plains (top row) and erosional *terra firme* (bottom row) substrates. We show data for canopy openings that extend to 1 m aboveground (left column) and 20 m aboveground (right column). The parameter *λ* is the scaling exponent for the power-law Zeta distribution fitted to the data using maximum likelihood (Appendix 1). The sample size (n) and flight block (FB) are also indicated. Note that flight block 11 contains only depositional substrates.

**
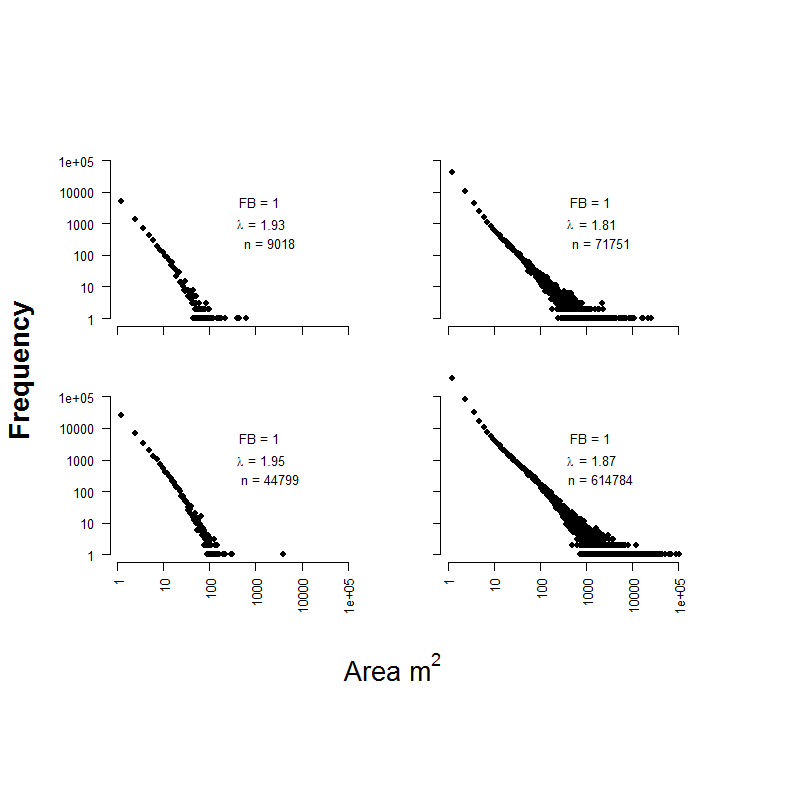

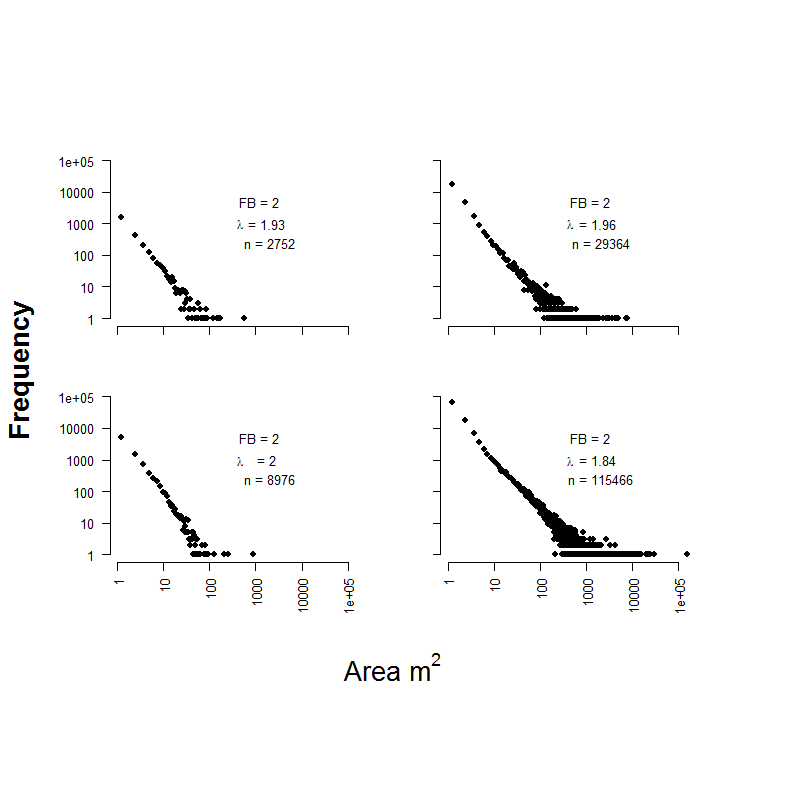

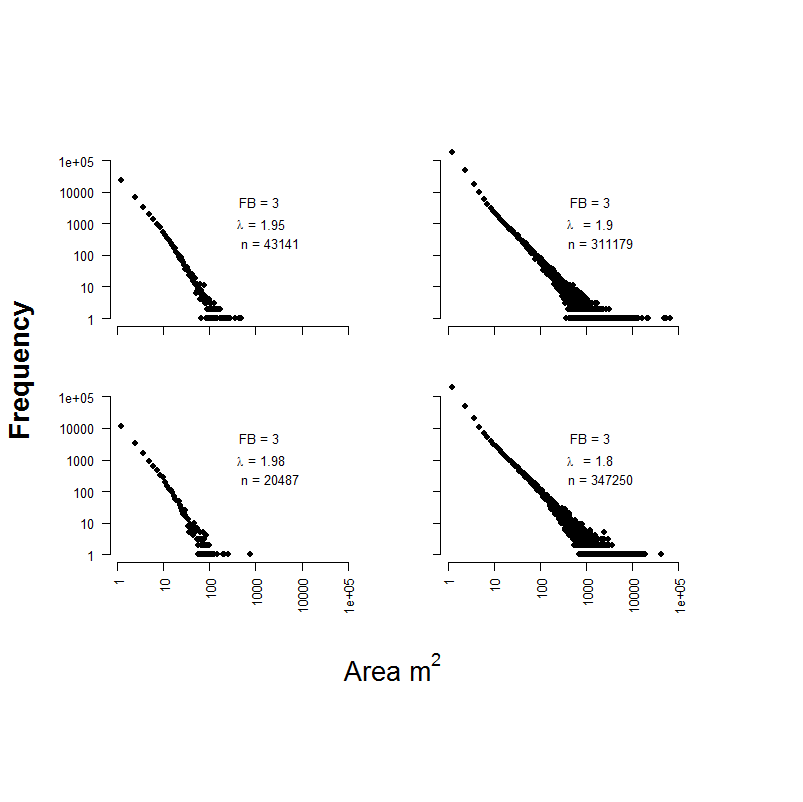

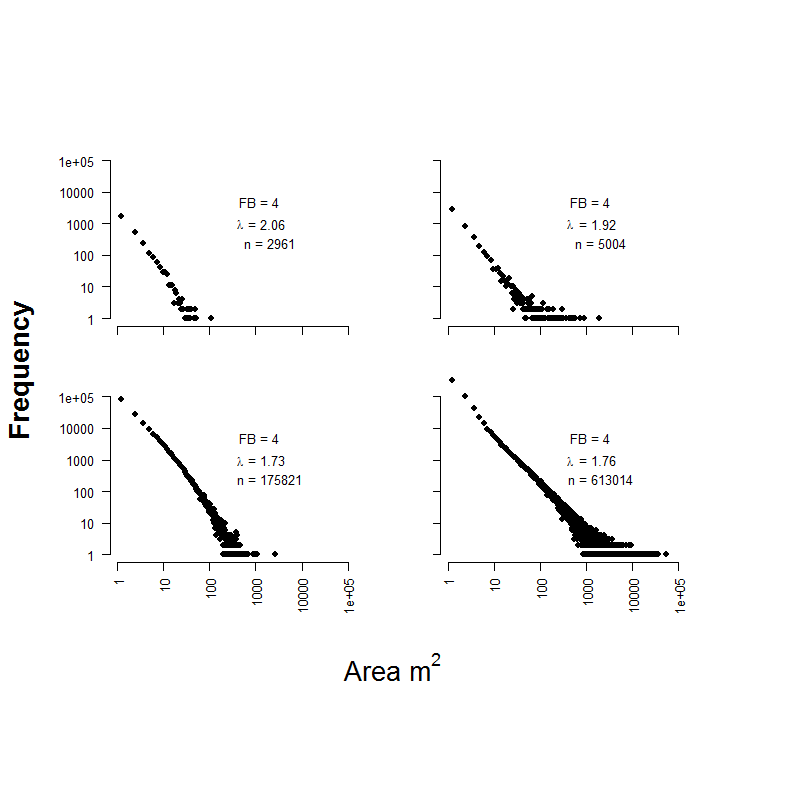

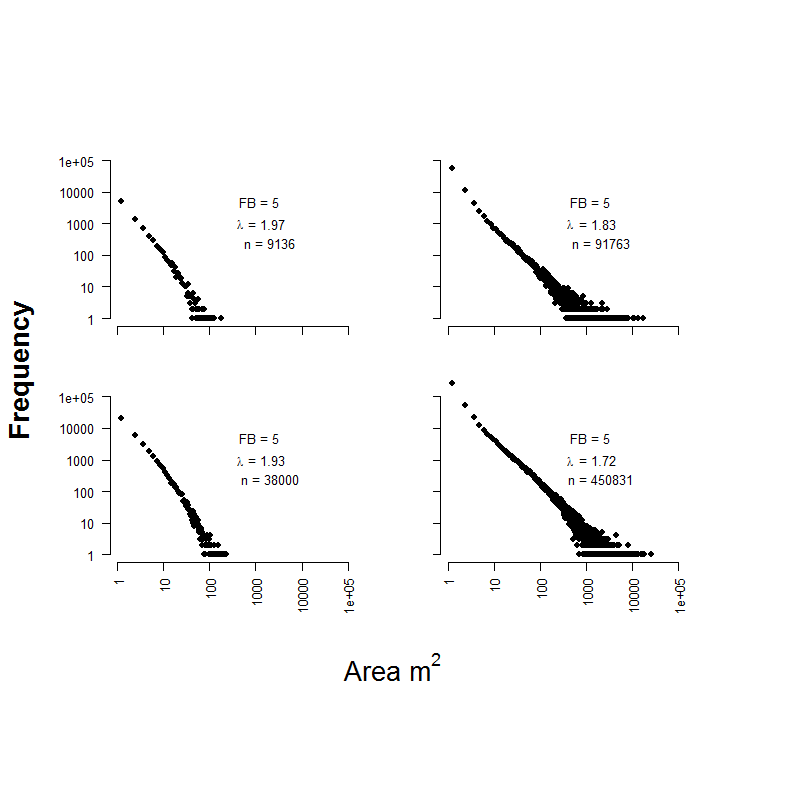

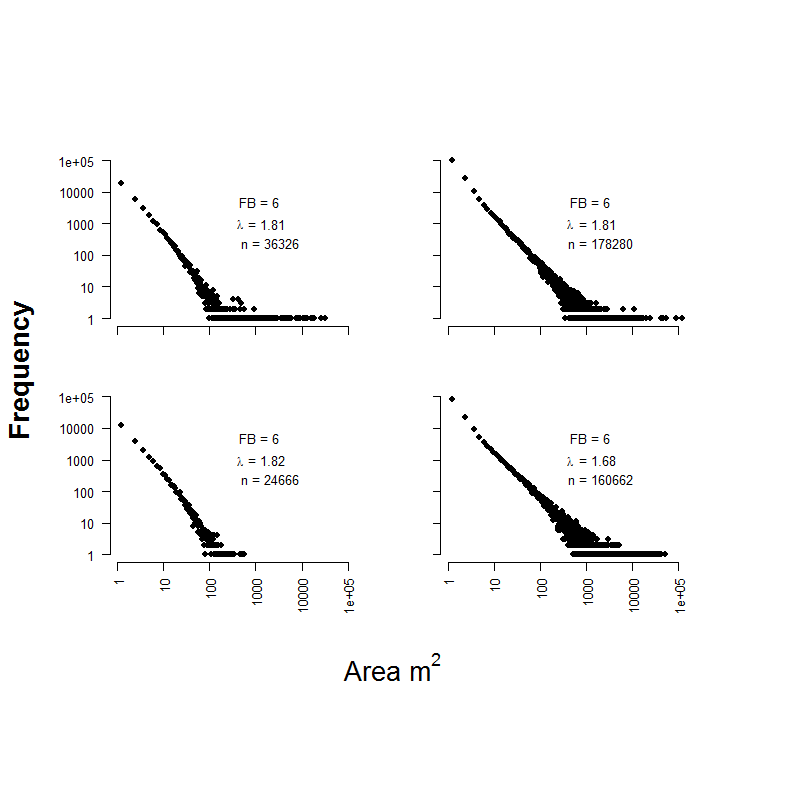

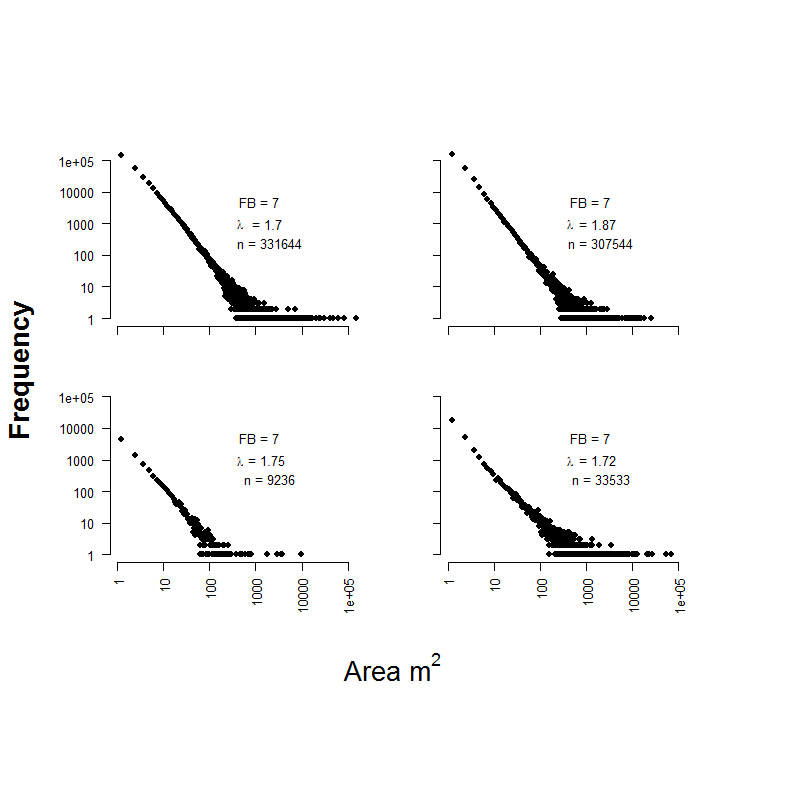

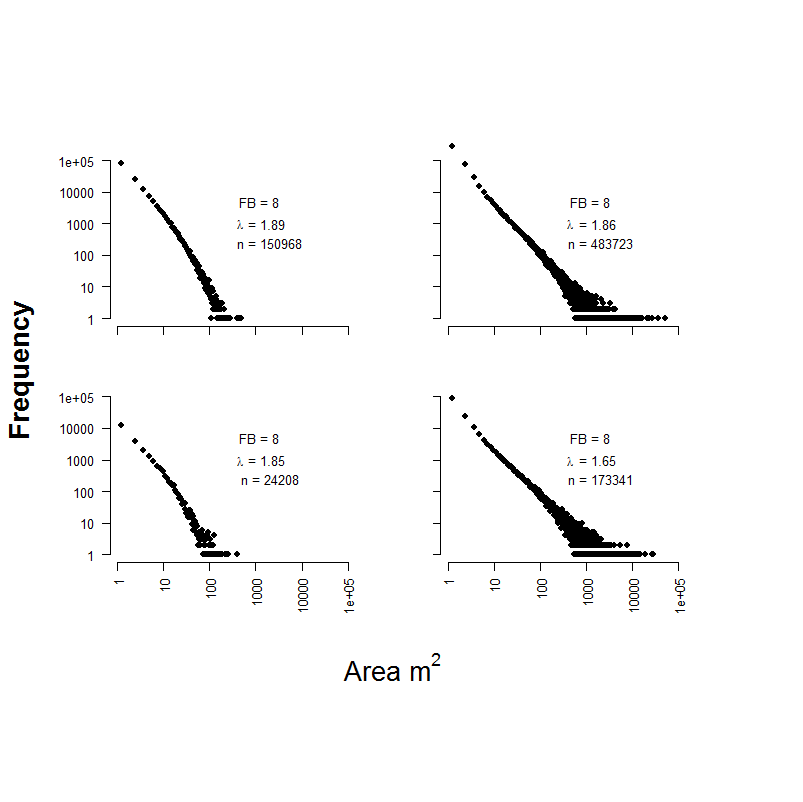

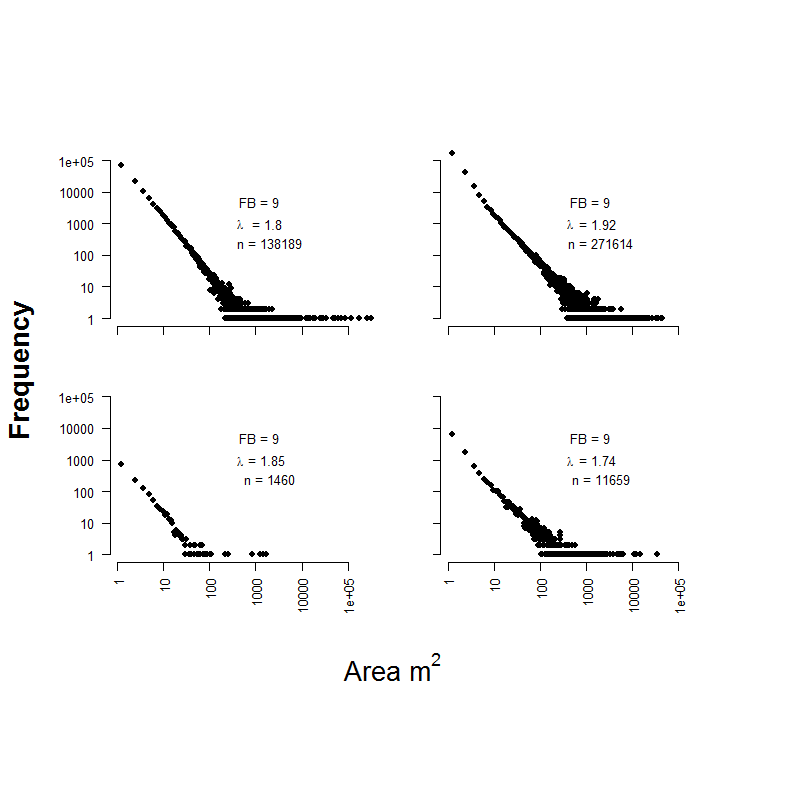

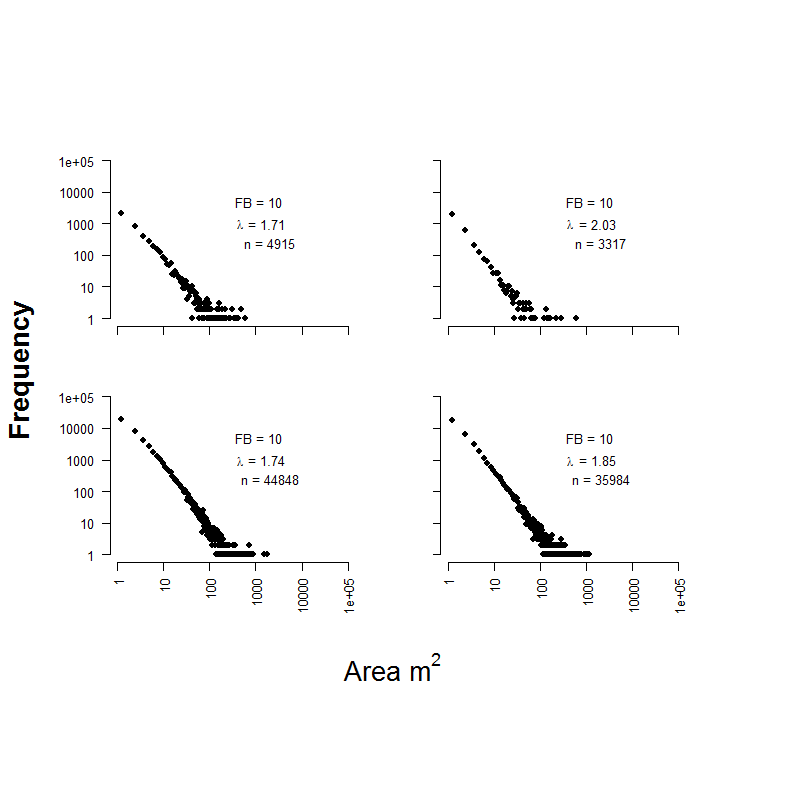

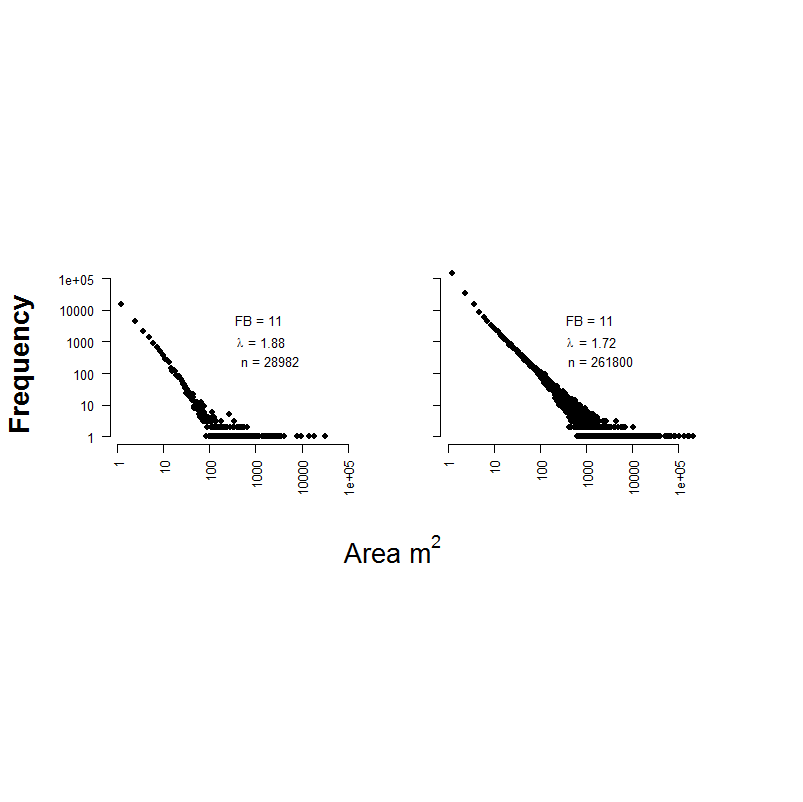

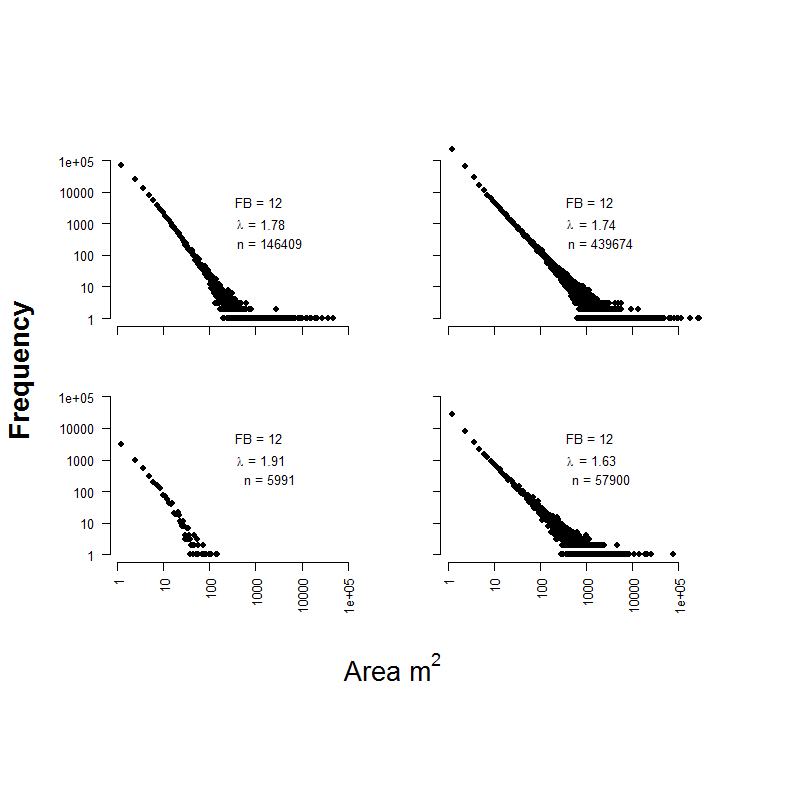

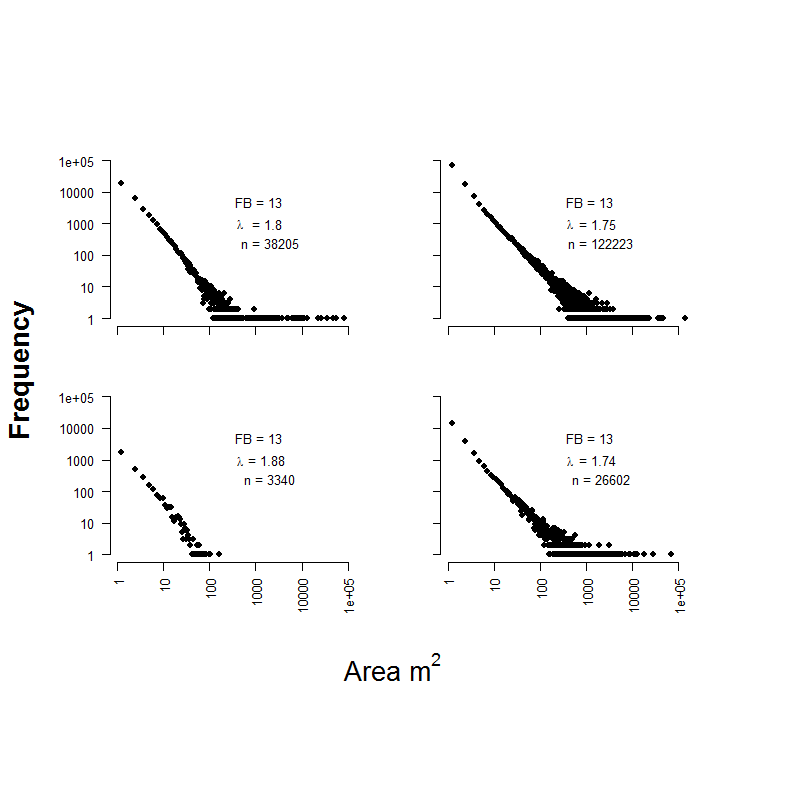
**
